# Difficult Biliary Stones: A Comprehensive Review of New and Old Lithotripsy Techniques

**DOI:** 10.3390/medicina58010120

**Published:** 2022-01-13

**Authors:** Edoardo Troncone, Michelangela Mossa, Pasquale De Vico, Giovanni Monteleone, Giovanna Del Vecchio Blanco

**Affiliations:** 1 Department of Systems Medicine, University of Rome Tor Vergata, 00133 Rome, Italy; troncone.edoardo@gmail.com (E.T.); michelangela.mossa@gmail.com (M.M.); gi.monteleone@med.uniroma2.it (G.M.); 2 Department of Anaesthesia, University of Rome Tor Vergata, 00133 Rome, Italy; devico.p@gmail.com

**Keywords:** endoscopic retrograde cholangiopancreatography, ERCP, cholangioscopy, electro-hydraulic lithotripsy, laser lithotripsy, spyglass, choledocholithiasis, hepatolithiasis, ESWL

## Abstract

Biliary stones represent the most common indication for therapeutic endoscopic retrograde cholangiopancreatography. Many cases are successfully managed with biliary sphincterotomy and stone extraction with balloon or basket catheters. However, more complex conditions secondary to the specific features of stones, the biliary tract, or patient’s needs could make the stone extraction with the standard techniques difficult. Traditionally, mechanical lithotripsy with baskets has been reported as a safe and effective technique to achieve stone clearance. More recently, the increasing use of endoscopic papillary large balloon dilation and the diffusion of single-operator cholangioscopy with laser or electrohydraulic lithotripsy have brought new, safe, and effective therapeutic possibilities to the management of such challenging cases. We here summarize the available evidence about the endoscopic management of difficult common bile duct stones and discuss current indications of different lithotripsy techniques.

## 1. Introduction

The presence of gallstones is a very common condition worldwide, especially in developed countries where it is estimated to affect 10–15% of adult population and accounts for a great part of total gastrointestinal-related healthcare expenditures [1,2]. Up to 25% of patients with gallbladder stones develop symptoms and/or complications, which can be severe in 1–2% of the cases [3,4,5]. Complications are mostly determined by the migration of stones into the common bile duct, which may result in biliary pain, pancreatitis, bile duct obstruction, and cholangitis (with significant morbidity). Endoscopic retrograde cholangiopancreatography (ERCP) is the cornerstone for therapy of common bile duct stones. Generally, after endoscopic biliary sphincterotomy, bile duct stones are extracted with balloon or basket catheters, which are considered “standard” techniques to achieve bile duct clearance. Randomized controlled trials (RCT) comparing such techniques for small bile duct stones (i.e., <11 mm) reported slightly better results for balloon over basket catheter, especially for stones <6 mm that could be difficult and time-consuming to grasp with a standard four wires basket [6,7]. Given the overall similar effectiveness, European guidelines suggest to use indifferently either technique for retrieving biliary stones, depending on the anatomy of the bile duct, shape and size of stones and personal preferences [8]. Whilst most cases are successfully treated with such first-line approaches, about 10–15% need alternative and/or adjunctive techniques to achieve bile duct clearance [9,10,11]. These conditions are generally defined as “difficult” bile duct stones, a broad category of cases that encompasses very different scenarios, including large or multiple stones, peculiar stone shapes (e.g., barrel-shaped), stones located above a stricture or impacted, intrahepatic stones, altered distal bile duct (oblique, narrowed, sigmoid-shaped), periampullary diverticula, surgically altered anatomy or specific patient’s general conditions [9,12]. It is obviously impossible to provide a single therapeutic strategy that could help to manage such large and heterogeneous group of possible cases. It is also clear that the experience and technological advances are changing the definition of “difficult” stones over time. In this complex scenario, the lithotripsy, that is the possibility to fragment the stones, has been largely used to overcome difficult cases secondary to large size of stones or challenging anatomical conditions of the bile duct. Mechanical lithotripsy with basket catheters is the most used lithotripsy technique due to its wide availability and effectiveness. When mechanical lithotripsy fails (for example excessive stone size or stone impaction), extracorporeal shock wave lithotripsy (ESWL) could represent an alternative, with conflicting results in terms of efficacy and safety [8]. Since these techniques has been available for a long time, they could be considered as “traditional” or “old” lithotripsy techniques. Finally, the groundbreaking innovations related to cholangioscopy and the possibility to perform intraductal lithotripsy, that means the “new” cholangioscopy-assisted lithotripsy techniques with laser or electro-hydraulic lithotripsy (EHL) systems, have markedly improved the management of several difficult cases. 

In this review, we will analyze different lithotripsy techniques for difficult bile duct stones, and will discuss unresolved questions regarding the therapeutic algorithm to adopt in such complex cases. 

## 2. Mechanical Lithotripsy

**Figure 1:**
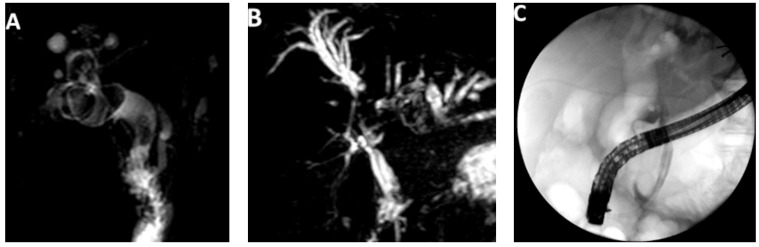
Cases of difficult biliary stones not amenable to conventional ERCP treatment. (**A**,**B**) Magnetic Resonance Images showing complex bile duct stones with large signal defects extending to the hilum and intrahepatics; (**C**) cholangiogram showing a very large common bile duct stone. All cases were successfully managed with single-operator cholangioscopy-assisted electrohydraulic lithotripsy.

**Figure 2:**
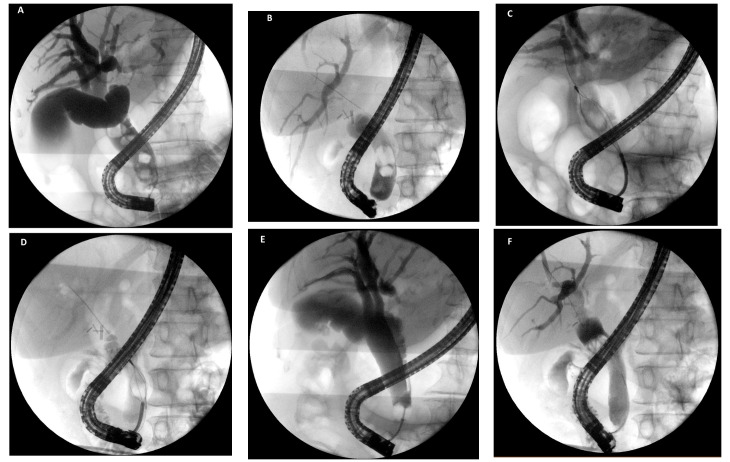
(**A**,**B**) cholangiograms showing multiple and large common bile duct stones; (**C**,**D**) fluoroscopy showing mechanical lithotripsy with basket of the largest stones; (**E**,**F**) final occlusive cholangiograms showing complete clearance of the bile duct after lithotripsy and extraction of fragments.

Stone size is a well-recognized factor that can influence the success of the procedure and, in particular, size above 15 mm has been identified as a risk factor for difficult extraction [13,14] (Figure 1). Other factors that can associate with difficult or incomplete stone extraction are summarized in Table 1. Mechanical lithotripsy consists in the fragmentation of the stone with a mechanical lithotripter to facilitate its removal from the biliary tract. A mechanical lithotripter includes a reinforced wire basket, which is used to grasp the stone into the bile duct, a metal sheath, and a handle, by which the basket with the entrapped stone is retracted towards the metal sheath, thus exerting a crushing force that fragments the stone [15]. Two main types of mechanical lithotripsy exist: through-the-scope (TTS) lithotripsy and out-of-the-scope (OTS) lithotripsy. TTS lithotripsy is generally performed with integrated devices that incorporate all components of the system and work through the working channel of the duodenoscope; it is generally used for elective procedures (Figure 2). OTS lithotripsy is performed with a metal sheath and a handle, which are applied to a non-lithotripter basket entrapped in the bile duct after grasping the stone, and therefore is used as a “salvage” procedure [8,15]. To perform salvage lithotripsy, the basket handle is cut off and the plastic sheath covering the wires is removed. Then, the metal sheath is advanced over the wires of the impacted basket under fluoroscopic guidance to the level of the stone, and a dedicated lithotripsy handle is attached to the metal sheath and the basket wires. The retraction of the basket against the metal sheath determines the fragmentation of the stone or the breakage of the basket, thus finally allowing a safe extraction of the system. Mechanical lithotripsy is reported to be an effective and safe procedure, with a success rate ranging between 76% and 91% [16,17,18,19,20,21,22]. However, the success rate at the first attempt is about 50–70%, and therefore a significant proportion of patients need repeated procedures. Observational studies investigating predictive factors of mechanical lithotripsy failure identified the size of the stone, especially in relation to the caliber of the duct, as a major factor influencing the success [17,19]. A retrospective study analyzing 134 patients who underwent mechanical lithotripsy for difficult stones reported an overall success rate of 76.1% [23]. Multivariate logistic regression analysis showed that stone impaction, size above 30 mm, and stone size/bile duct diameter ratio >1.0 were significant predictors of lithotripsy failure, with estimated odds ratios of 17.83, 4.32 and 5.47, respectively. Consistently, stone impaction hinders the progression of a basket proximal to the stone or prevents the fully open of the basket and thus the capture of the stone.

Mechanical lithotripsy is generally safe, and most of adverse events (AE) reported are secondary to biliary cannulation and therefore are commonly considered as ERCP-related AE. The most feared mechanic lithotripsy-related AE is the entrapment of the basket within the bile duct or a disfunction of components of the lithotripter system (e.g., broken wires/baskets or handle) that resulted in trapped basket. A retrospective study including 643 cases of biliary mechanical lithotripsy reported 29 lithotripsy-related AE (i.e., 11 cases of trapped/broken baskets, 8 traction wire fracture, 7 broken handle and 3 perforation or duct injury) in 23 patients (3.6%) [24]. Importantly, almost all AE were treated endoscopically with salvage or alternative lithotripsy techniques, extension of sphincterotomy or stent placement, while only one case required surgery [24].

In the last years, endoscopic papillary large balloon dilation (EPLBD) has been largely adopted to manage large common bile duct stones [25]. EPLBD is usually performed after limited sphincterotomy with a balloon sized 12–20 mm, at a target size not exceeding the upstream bile duct caliber in order to avoid perforation, and for a duration between 30 to 60 seconds from the disappearance of the balloon waist [8,26]. Several randomized trials have investigated the efficacy of EPLBD with or without sphincterotomy compared to sphincterotomy alone [27,28,29,30,31]. A meta-analysis including 902 patients from 7 studies reported no significant differences in terms of overall stone clearance (98% vs. 95%, RR 1.01 [0.97, 1.05]; *p* = 0.60) and stone clearance at the first session (87% vs. 79%, RR 1.11 [0.98, 1.25]; *p* = 0.11) [32] between EPLBD with limited sphincterotomy and sphincterotomy alone. However, compared to sphincterotomy alone, EPLBD with limited sphincterotomy reduced need for mechanical lithotripsy (15% vs. 32%; RR 0.49 [0.32, 0.74]; *p* = 0.0008) and was associated with fewer AE (11% vs. 18%; RR 0.58 [0.41, 0.81]; *p* = 0.001) [32]. These results were in line with a meta-analysis of 6 randomized controlled trials (RCT) involving 835 patients and reporting no significant differences between the two procedures in terms of complete stone removal, stone removal in the first session, AE and procedure time. The study highlighted reduced need for mechanical lithotripsy (OR 0.26, 95% CI: 0.08–0.82, *p* = 0.02), especially in patients with a stone size larger than 15 mm (OR 0.15, 95% CI: 0.03–0.68, *p* = 0.01) [33]. The efficacy of EPLBD in decreasing mechanical lithotripsy has been confirmed in further recent RCTs [28,31,34,35,36]. Furthermore, a recent multicenter RCT from Kogure and colleagues compared the efficacy and safety of EPLBD alone vs. sphincterotomy for the removal of large (≥10 mm) stones, and confirmed that EPLBD, even without sphincterotomy, achieved a significantly higher rate of complete stone removal in a single session compared with sphincterotomy alone (90.7% vs. 78.8%; *p* = 0.04), with fewer mechanical lithotripsy (30.2% vs. 48.2%; *p* = 0.02) [37]. Strikingly, no differences in AE (9.3% vs. 9.4%)—and specifically in post-ERCP pancreatitis (4.7% vs. 5.9%)—were noted between the groups. Mechanical lithotripsy is certainly a valuable tool in the management of large stones, but some potential issues are associated with this technique. Cannulation of the bile duct with a through-the-scope lithotripter could be challenging if a non-wire-guided device is used. Opening the basket and capturing the stone could be difficult and time-consuming, especially for larger stones in smaller bile ducts. There is an obvious need for multiple and repeated duct sweeping to remove fragments after stone crashing, and if adequate duct clearance is not achieved, a stent or naso-biliary drainage should be placed, with the subsequent need for further procedures and increased patient’s discomfort. Bearing this in mind, the availability of a relatively easy, safe and effective tool as EPLBD, which potentially allows to extract “en-bloc” large stones after 30–60 seconds of balloon inflation is obviously attractive, especially considering that solid data from RCTs and meta-analysis have demonstrated that mechanical lithotripsy could be avoided in a significant proportion of cases. Consistently, European guidelines suggest limited sphincterotomy with EPLBD as first-line therapeutic approach if large bile duct stones are seen on cholangiography or cross-sectional images [8].

Temporary stent placement to provide duct drainage should be always considered as alternative strategy when approaching difficult stones, especially if the operative conditions do not allow complex and/or prolonged procedures, due to insufficient technical support (e.g., lack of dedicated devices for lithotripsy or cholangioscopy), insufficient endoscopist’s expertise, or if patient’s condition requires a rapid biliary decompression (i.e., severe cholangitis) [8]. Several data reported a significant reduction of stone size after a variable time of indwelling biliary stents [38,39,40,41,42,43]. The hypothesized mechanism is the continuous friction between the stone and plastic stents that produces mechanical stress and results in changing of size and/or number of stones [40]. Data on stone removal rates after temporary stenting mostly come from retrospective studies, and range from 44% to 96%, but are frequently about 90% or above [39,40,42,43,44]. A recent retrospective study including 85 patients who underwent plastic biliary stent placement for difficult stones reported that 7-Fr rather than 10-Fr plastic stents associated with complete clearance in multivariate analysis [45]. However, the overall complete stone clearance rate was quite low (64.7%, 55/85) at the second ERCP, and still about 28% of patients required mechanical lithotripsy [45]. Although prospective comparative data are lacking, one could speculate that stone size reduction after stenting may decrease the need for mechanical lithotripsy at subsequent ERCP, but at the expenses of the need for repeated procedures and possible increased costs. Therefore, complete clearance should be attempted in one procedure when feasible and safe, and temporary stenting should be chosen after case-by-case evaluation.

## 3. Extracorporeal Shock Wave Lithotripsy

Extracorporeal shock wave lithotripsy (ESWL) was originally developed to treat urologic stones, and then proposed for biliary and pancreatic lithiasis. Outside the body, the lithotripter generates shock waves that are transmitted through the tissues within the body to fragment the target stones [15]. The energy needs to be precisely focused on the stones for effective lithotripsy and to avoid damage to the surrounding tissues. Biliary ESWL is a complex procedure, which needs the placement of a biliary stent or naso-biliary drainage before the lithotripsy to allow fluoroscopic targeting of the stones and to provide continuous saline irrigation; furthermore, ERCP is needed after lithotripsy to extract the fragments. According to a recent meta-analysis, which included 32 studies with 1969 patients undergoing EHL (*n* = 277), laser lithotripsy (*n* = 426) or ESWL (*n* = 1266) for difficult biliary stones, ESWL had the lowest complete ductal clearance rate (84.5%) compared to laser lithotripsy (95.1%) and EHL (88.4%, *p* < 0.001) [46]. Moreover, RCTs have shown that ESWL requires more sessions to achieve complete duct clearance compared to intraductal laser lithotripsy [47,48]. ESWL-related AE include pain, hemobilia, cholangitis, sepsis, pancreatitis, and hematuria, and have been reported in up to 14% of patients [15,49,50]. Owing to technical and logistic difficulties, ESWL is currently limited to difficult biliary stones refractory to conventional treatments, when intraductal lithotripsy is not available [8].

## 4. Cholangioscopy-Assisted Lithotripsy: The “New” Lithotripsy Technique

**Figure 3:**
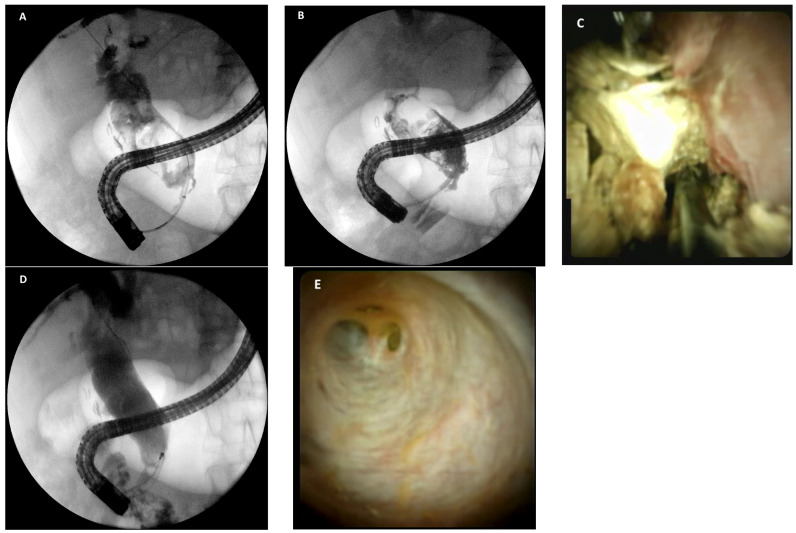
(**A**) cholangiogram showing a large common bile duct stone; (**B**) fluoroscopic image of the cholangioscope in the common bile duct during lithotripsy; (**C**) cholangioscopic view of intraductal lithotripsy with electro-hydraulic probe; (**D**) final cholangiogram showing clearance of the bile duct after lithotripsy and extraction of fragments; (**E**) cholangioscopy showing clearance of the bile duct.

The recent technological evolution of cholangioscopes and their widespread adoption has greatly improved the management of several complex bilio-pancreatic disorders. The first direct peroral cholangiopancreatoscopy was described in 1975, and, since then, several efforts have been made to move from an “indirect” radiological toward a “direct” endoscopic visualization of the biliary tree. These efforts finally led to the development of high-quality video cholangioscopes and dedicated devices to perform diagnostic and therapeutic intra-ductal procedures [51]. Three cholangioscopy techniques are traditionally described: “direct” peroral technique, which generally uses ultrathin gastroscopes to directly access the bile duct, even if also standard gastroscopes could be used in selected cases [52]; “dual-operator” mother-baby technique, which uses dedicated cholangioscopes that are introduced through the working channel of the duodenoscope; “single-operator” mother-baby technique, in which the cholangioscope and the duodenoscope can be managed by a single endoscopist [51]. Direct cholangioscopy is technically demanding, and in many cases does not allow diagnostic or therapeutic interventions in the intrahepatic ducts. Moreover, severe AE, especially air embolism, have been reported, and therefore this procedure should be carried out under CO_2_ insufflation [53,54,55]. On the other hand, “dual-operator” mother-baby cholangioscopy suffers from the limitations related to the need for two operators, and to the cost and fragility of the equipment, despite good results in terms of efficacy [56,57]. Cholangioscopy progressively spread since the advent of the SpyGlass direct visualization system, a single-operator cholangioscopy (SOC). This system was introduced by Boston Scientific (Natick, Massachusetts, USA) and then improved with the development of the SpyGlass Digital-Imaging System (D-SOC) [58]. The platform consists of two main components: a sterile, single-use access catheter (10.5 Fr SpyScope^TM^ Catheter) and the SpyGlass DS digital controller (the processor) with light-emitting diode (LED) illumination source [51,59]. The D-SOC has two irrigation channels and one 1.2 mm diameter working channel for aspiration and accessory devices, such as lithotripsy probes. Two cholangioscopy-assisted lithotripsy techniques are available: electro-hydraulic lithotripsy (EHL) and laser lithotripsy. EHL systems work through a bipolar probe and a charge generator; transmitting a charge across the electrodes at the tip of the probe generates a spark. This determines expansion of the surrounding fluid and finally results in an oscillating shock wave of pressure that fragment the stones [15]. Under direct endoscopic visualization, the probe is directed at the stone and is advanced at least 5 mm from the tip of the cholangioscope, positioned 1 to 2 mm from the stone [60]. When a correct and stable position is achieved, and the stone is clearly visualized, the EHL system is activated by a foot pedal. Saline solution irrigation is crucial to provide a medium for shock wave transmission, as well as to allow visualization of the duct and stones and to flush away debris (Figure 3). Autolith Touch EHL (Nortech; Northgate Technologies Inc., Elgin, Ill, USA) is the EHL system approved by the U.S. Food & Drug Administration (FDA) for biliary stones, and allows selection between differ power settings (low, medium and high-power) and number of pulses delivered by a single foot pedal activation [15]. Laser lithotripsy systems work by focusing laser light of a high-power density on the surface of a stone. The concentrated high power creates a plasma composed of a gaseous collection of ions and free electrons that oscillates and induces waves fracturing the stone surface [15]. Holmium:yttrium aluminum garnet (YAG) lasers are commercially available (Lumenis Inc., San Jose, CA, USA), and have been approved from FDA for the treatment of gallbladder and bile duct stones, after being largely used for urinary tract stones. The laser-light wavelength is in the near-infrared spectrum (2100 nm) and delivers high-energy pulses of about 500 to 1000 mJ through delivery fibers that fit through the working channels of most cholangioscopes [61,62]. Although direct visualization is strongly suggested when using these lithotripsy techniques, both EHL and laser probes have been used without cholangioscopy under fluoroscopy guidance alone [61,63]. After lithotripsy, the stone fragments are subsequently extracted with standard techniques. However, multiple and small fragments resulting from lithotripsy can impact in small bile ducts or cystic duct due to the continuous saline flushing. Therefore, dedicated basket working through the small channel of the cholangioscope has been developed to manage special situations [64,65]. Intraductal lithotripsy, either with EHL or laser, can be performed during all types of cholangioscopy. Observational studies on cholangioscopy-assisted lithotripsy with mother-baby dual-operator system reported a success rate ranging from 77% and 96%, and the need for repeated procedures in up to 25–50% [66,67,68]. A meta-analysis from Korrapati and colleagues on diagnostic and therapeutic cholangioscopy included 31 studies and more than 2000 patients who underwent different cholangioscopy techniques with intraductal lithotripsy. It was reported an overall estimated stone clearance rate of 88% (95% CI 85–91%) and an estimated stone recurrence rate of 13% (95% CI 7–20%), without significant evidence of heterogeneity among the studies [69]. Interestingly, meta-regression identified a significant association between SOC and technical success rates, with SOC demonstrating higher technical success rates (*p* < 0.01) [69]. Similarly, a recent large meta-analysis specifically targeted at cholangioscopy-assisted lithotripsy (35 studies involving 1762 patients), reported an overall stone fragmentation success of 91.22% (95% CI 88.14–93.56; I^2^ = 63.16%) with an average of 1.32 ± 0.62 lithotripsy sessions performed and a complete single-session success rate of 76.86% (95% CI 71.55–81.44; I^2^ = 74.33%) [70]. A RCT from Buxbaum et al. randomized 60 patients with stones >10 mm to SOC-assisted laser lithotripsy (42 patients) or conventional therapies (18 patients), the latter including balloon/basket extraction, mechanical lithotripsy and papillary dilation [71]. Endoscopic stone clearance was successful in 93% of patients undergoing cholangioscopy compared to 67% using conventional therapy only (*p* = 0.009). However, the study was limited by a small sample size and by a reduced and heterogeneous use of papillary dilation [71]. A subsequent large, prospective, single-arm, multicenter study from Maydeo and colleagues reported data on SOC-guided lithotripsy for difficult bile duct stones, defined as one or more of largest stone diameter ≥15 mm, failed prior attempt at stone clearance, impacted, multiple, hepatic duct location, or located above a stricture [72]. The Authors enrolled 156 patients who underwent 174 sessions of SOC-guided electrohydraulic or laser lithotripsy. SOC-guided stone clearance was achieved in a single procedure in 125/156 patients (80%, 95% CI 73–86%), and the overall success rate was 87%. No significant differences were observed in terms of rate of stone clearance in the first procedure using EHL or using laser lithotripsy (82%, 96/117 vs. 74% 29/39; *p* = 0.35). Stone clearance was significantly more likely in a single SOC-guided lithotripsy procedure when the largest stone was less than 30 mm in size, and generally the likelihood of successful stone clearance in a single procedure was higher for smaller sized stones [72].

No study has yet compared laser lithotripsy and EHL, and retrospective studies suggest similar success rate [73,74]. However, a meta-analysis suggests that laser lithotripsy had a higher complete ductal clearance rate (95.1%) compared with EHL (88.4%) and ESWL (84.5%; *p* < 0.001), together with a higher stone fragmentation rate (92.5% vs. 75.5% vs. 89.3%; *p* < 0.001) [46]. Similarly, meta-analysis from McCarty and colleagues showed a single session efficacy significantly higher for laser lithotripsy (82.97% vs. 70.85%; *p* = 0.021), and a mean procedure time significantly shorter compared to EHL, without differences in overall fragmentation rates (92.79% vs. 90.14%; *p* = 0.384) [70]. Results from a large, international, retrospective multicenter study including 407 patients who underwent D-SOC for difficult biliary stones reported similar overall technical success rate for EHL and laser lithotripsy (96.7% vs. 99%; *p* = 0.31), although a non-statistically significant trend in favor of laser was noted for single session clearance rate (74.5% by EHL vs. 86.1% by laser; *p* = 0.20) [75]. Interestingly, the same study highlighted a mean procedure time longer in the EHL group (73.9 min) compared to laser lithotripsy group (49.9 min; *p* < 0.001) [75]. At the present time, a laser lithotripsy system is available in only few endoscopy units. Therefore, guidelines suggest that the type of lithotripsy should depend on local availability and expertise, without supporting one method over the other [8,76,77]. 

**Figure 4:**
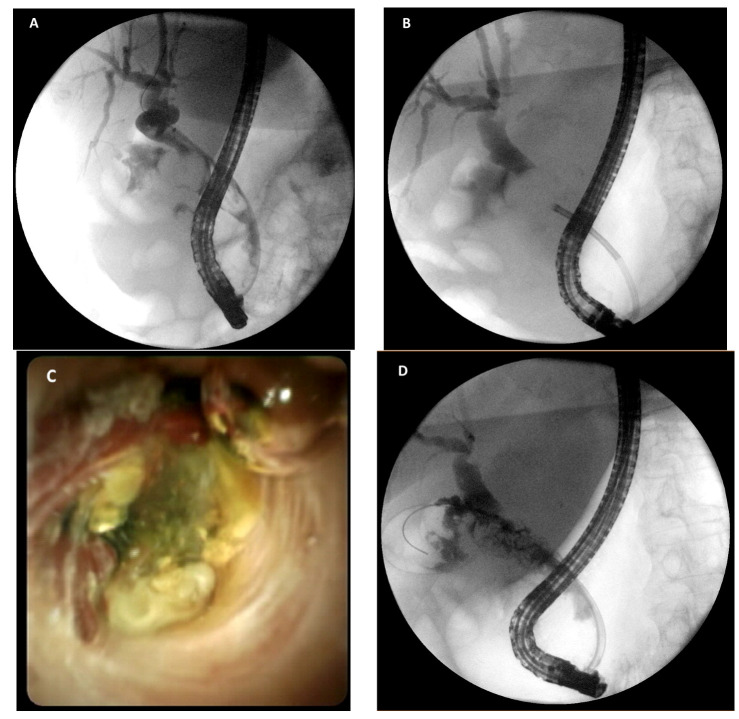
(**A**) cholangiogram showing a large stone impacted at the cystic duct insertion, obstructing the common bile duct; (**B**) fluoroscopic image of the cholangioscope approaching the impacted stone; (**C**) direct visualization of the impacted stone and inflamed biliary mucosa during cholangioscopy; (**D**) fluoroscopic image of the fragmented stone after electrohydraulic lithotripsy.

A condition that may particularly benefit from cholangioscopy-assisted treatment is Mirizzi’s syndrome, a common hepatic duct obstruction caused by extrinsic compression from an impacted stone in the cystic duct or infundibulum of the gallbladder [78]. Surgery is often complex, with significant rate of AE, and endoscopic clearance is challenging due to the frequent inability to pass or capture the impacted stone [79,80,81]. For such reasons, direct visualization and intraductal lithotripsy is considered as a high effective mini-invasive treatment, with high rate of technical success (Figure 4) [82,83,84,85,86].

Overall, cholangioscopy is a safe procedure and no difference in overall frequency of AE between intraductal and conventional therapy has been reported [76]. The overall AE rate reported in the meta-analysis from Korrapati et al. is 7%, ranging from 5% and 25% in the analyzed studies [69]. AE include pancreatitis, cholangitis, perforation, and other AE (e.g., ductal damage) at a rate of 2% (95%CI 2–3%), 4% (95%CI 3–5%), 1% (95%CI 1–2%), and 3% (95%CI 2–4%), respectively. The estimated rate of severe AE was 1% (95%CI 1–2%) [69]. While the risk of post-ERCP pancreatitis seems to be reduced in such patients, probably due to a high rate of previous sphincterotomy, cholangitis is the most reported and significant AE, and this could be related to prolonged intraductal irrigation during the procedure. Consistently, antibiotic prophylaxis is recommended in all patients who undergo cholangioscopy [8].

## 5. Percutaneous Bile Duct Stones Treatment

Cholangioscopy-assisted laser lithotripsy or EHL can be also performed through a percutaneous approach. This technique may be particularly useful for complex cases of intrahepatic stones (i.e., hepatolithiasis), or surgical altered anatomy. In the latter case, enteroscopy-assisted ERCP is often the first approach where expertise is available. However, the technique is challenging, burdened with the risk of small bowel perforation, and associated with heterogeneous success rate [87,88,89]. Furthermore, cholangioscopy is not possible through the enteroscope due to the length of the scope. Hepatolithiasis is often secondary to intrahepatic pathological conditions as postoperative biliary strictures, primary sclerosing cholangitis, progressive familial intrahepatic cholestasis or recurrent pyogenic cholangitis [90,91,92,93]. Percutaneous cholangioscopy-assisted management of complex hepatolithiasis has been reported effective in up to 80–97% of cases in old series, but the need for repeated treatment treatments was frequent, as well as the stone recurrence rate [91,94,95,96,97,98,99,100]. A retrospective study of 245 patients who underwent percutaneous transhepatic cholangioscopic lithotomy for hepatolithiasis reported a complete clearance rate of 85,3%, and a rate of major complications was 1.6% (two cases of liver laceration, one intra-abdominal abscess, one disruption of the percutaneous transhepatic biliary drainage) [101]. Notably, long-term follow-up showed that the overall recurrence rate of hepatolithiasis and/or cholangitis was 63.2%, and the incidence of recurrent cholangitis or cholangiocarcinoma was significantly higher in those who underwent incomplete treatment [101]. More recently, percutaneous cholangioscopy with the D-SOC system (SpyGlass™ DS) has been described, and cases of choledocholithiasis have been successfully treated with cholangioscopy-assisted lithotripsy, mostly in patients with surgical altered anatomy [102]. The system has further evolved with the recent development of a novel short D-SOC system specifically designed for percutaneous access, which not only maintains the features of the single-use digital cholangioscope, but also improves the deflection capability of the endoscope’s tip and offers improved handling owing to the shorter length of the endoscope (65 cm) [103]. Despite these exciting technological innovations, it should be kept in mind that serious AE can occur in up to 6.8–10.9% of patients after percutaneous radiological stone extraction, as reported in recent large series, and further studies are needed to define which specific clinical situations and patients will mostly benefit from this approach [104,105]. Therefore, it is currently recommended that percutaneous radiological stone extraction should be reserved for the patients in whom conventional transpapillary techniques fail or are not possible [8,77]. 

## 6. Different Approaches for Management of Difficult Biliary Stones

The above-mentioned studies highlight that several approaches are currently available to manage endoscopically difficult biliary stones. These approaches may need specific technological equipment and different level of expertise. Moreover, since efficacy and safety data mostly come from observational studies and there are few comparative studies, it remains to clearly define which technique should be preferred and for which patients (Table 2). Most of the available RCT comparing cholangioscopy-assisted lithotripsy with conventional techniques clearly showed higher success rate of cholangioscopy [71,106,107,108]. In particular, Bang et al. recently compared SOC-assisted laser lithotripsy and EPLBD and confirmed a significantly higher success rate success for SOC (93.9% vs. 72.7%; *p* = 0.021) [106]. Similarly, SOC lithotripsy performed better than mechanical lithotripsy even after failed EPLBD [107]. Facciorusso and colleagues aimed at overcoming the paucity of comparative studies on large bile duct management, and performed a network meta-analysis of RCT, including and comparing all of the available approaches (i.e., sphincterotomy, balloon sphincteroplasty, sphincterotomy plus EPLBD, mechanical lithotripsy and SOC-assisted lithotripsy) [109]. By analyzing 19 RCT involving 2752 patients, the Authors showed that all treatments except mechanical lithotripsy significantly outperformed sphincterotomy in terms of stone removal rate (RR, 1.03–1.29), and cholangioscopy ranked the highest in increasing the success rate of stone removal (SUCRA score, 0.99) followed by sphincterotomy plus EPLBD (SUCRA score, 0.68). Notably, included RCT had different inclusion criteria and heterogeneous definition of difficult stones, therefore caution is needed when interpreting such results. Strikingly, SOC and sphincterotomy plus EPLBD outperformed the other approaches when only studies reporting on stones greater than 15 mm were taken into consideration. Safety analysis showed similar AE rate [109]. 

Do all of these data indicate that cholangioscopy is the best solution for every case of difficult biliary stones? The question is more complicated than it might seem. Despite the evident advantages, the following aspects should be considered when planning a SOC-assisted treatment. First, the technical equipment (cholangioscopy system, lithotripter, probes) and the expertise are not available in all endoscopy units, and therefore deciding to treat biliary stones with SOC may mean to refer the patients to other centers. Second, the procedure is burdened with high costs. While one could argue that the increased costs would be balanced by the reduced number of ERCP performed with other ineffective techniques, definitive cost-effective studies still lack. The economic impact of SOC in management difficult stones was evaluated using a decision-tree model in a work from Deprez and colleagues [110]. In this model, SOC-assisted lithotripsy was introduced in the algorithm after one failed conventional ERCP (overall failure rate at first procedure was set at 31%), and this strategy determined a decrease in the number of procedures (−27% relative reduction) and costs (−€73,000; −11% relative reduction) when compared to the strategy based on repeated conventional ERCP [110]. Similarly, Alrajhi et al. performed a cost-effectiveness analysis investigating three possible strategies with a progressively delayed introduction of SOC in managing difficult stones after one failed ERCP. Again, the authors showed that the least expensive strategy was to perform SOC early after the first failed ERCP, while postponing SOC resulted in increased cost and marginal or no increase in efficacy [111]. Overall, these models suggest that early adoption of SOC could be cost-effective, reducing the number of procedures, costs and, not less important, minimizing patient’s stress subsequent to repeated procedures with incomplete treatment and/or AE. 

On the other hand, it should be also kept in mind that most data come from expert tertiary centers and may not be generalizable to all centers performing ERCP. In our experience, patients with complex choledocholithiasis who had undergone several ineffective ERCP and who also experienced AE secondary to long-term indwelling plastic stents (e.g., cholangitis, hepatic abscesses), have been successfully treated with one or two sessions of SOC-assisted lithotripsy during the same hospital admission. In our opinion, this further highlights the need of timely referral of complex cases to a tertiary center that can provide multiple therapeutic approaches, in order to maximize clinical success.

**Figure 5:**
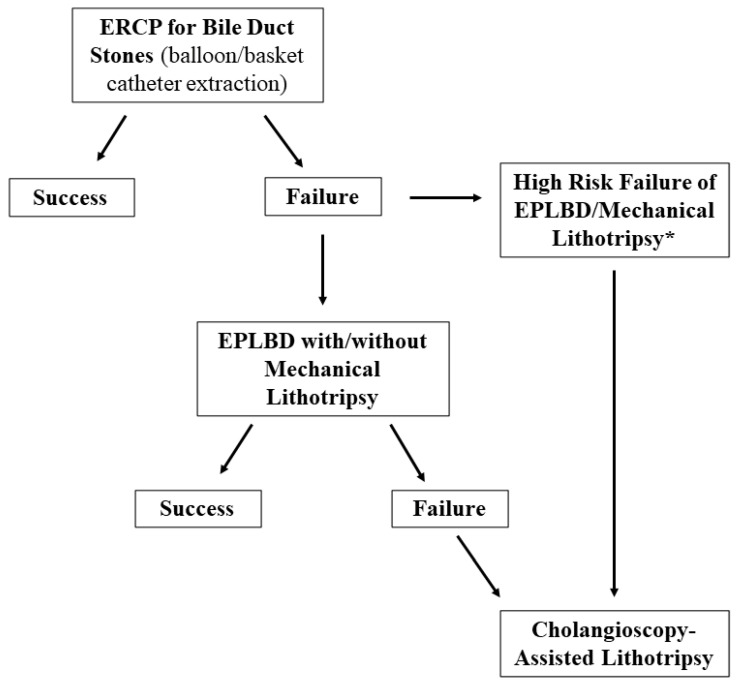
Flowchart for management of biliary stones. EPLBD, Endoscopic Papillary Large Balloon Dilation. * risk factors for failure of EPLBD or mechanical lithotripsy: stone impaction, size above 30 mm, stone size/extrahepatic bile duct ratio > 1, tapered bile duct.

Ideally, the definition of predictive factors of outcome could help to identify patients who may benefit from repeated conventional ERCP, and those who should go directly to SOC-assisted therapy or should be referred to expert centers. For instance, the stone size/extrahepatic bile duct ratio >1 and tapered bile duct have been associated with treatment failure with EPLBD, and thus such features, that can be often defined before the procedure with cross sectional images, could identify a sub-group of patients to be treated directly with cholangioscopy [106]. A flowchart for the management of difficult biliary stones is proposed in Figure 5.

## 7. Conclusions

Biliary stones are a very common indication for ERCP, and thus are frequently encountered by therapeutic endoscopists in daily practice. While representing a small part of the total volume of cases, difficult biliary stones could pose a relevant challenge and require advanced skills. Technological advances in biliary endoscopy have significantly enriched the therapeutic armamentarium to manage such conditions, thereby reducing the need for more invasive treatments and improving patient’s outcomes. However, when to choose a specific technique and in which patients still remains an open question. Further studies are needed to define the best therapeutic algorithm which maximizes clinical efficacy, safety, and cost-effectiveness. 

## Figures and Tables

**Table 1:** Factors possibly associated with difficult or incomplete stone extraction.

Stone-related Factors Size (>10 mm, >15 mm) Shape (e.g., barrel-shaped) Number (i.e., multiple) Impacted Stones (e.g., bile duct/stone disproportion, biliary strictures) Intra-hepatic stones
Bile Duct-related Factors Angulated Distal Bile Duct Tapered Distal Bile Duct Sigmoid-shaped Bile Duct Peri-diverticular Papilla
Post-Surgical Anatomy (e.g., long biliary limbs)
Patient’s Clinical Conditions (e.g., severe acute cholangitis)
Low Experienced Endoscopist
Inadequate Setting (e.g., out-of-hours ERCP with non-dedicated staff)

**Table 2:** Randomized controlled trials comparing different lithotripsy techniques. EPLBD, endoscopic papillary large balloon dilation; SOC, single-operator cholangioscopy; EHL, electrohydraulic lithotripsy; LL, laser lithotripsy; CBD, common bile duct.

Author, Year [Ref.]	No. of Centers	Techniques	Definition of Difficult Stones	No. of Patients	Success Rate	*p*	Adverse Events
Stefanidis et al., 2011 [112]	1	Sphincterotomy + EPLBD vs. Mechanical Lithotripsy	Large > 12 mm	45 45	44/45 (97.7%) 41/45 (91.1%)	0.36	2/45 (4.4%) 9/45 (20%)
Franzini et al., 2017 [108]	1	SOC-EHL vs. Sphincterotomy + EPLBD	Multiple (>10), large >15 mm, above strictures, disproportion CBD/stone > 2 mm	50 50	37/48 (77.1%) 36/50 (72%)	>0.05	2/48 (4.2%) 6/50 (12%)
Buxbaum et al., 2017 [71]	1	SOC-LL vs. Conventional Therapies	Large > 10 mm	42 18	39/42 (92.9%) 12/18 (66.7%)	<0.01	4/42 (9.5%) 2/18 (11.1%)
Angsuwatchakaron et al., 2019 [107]	2	SOC-LL vs. Mechanical Lithotripsy	Failed extraction after Sphincterotomy + EPLBD	16 16	16/16 (100%) 10/16 (63%)	0.009	2/16 (12.5%) 1/16 (6.3%)
Bang et al., 2020 [106]	1	SOC-LL vs. Sphincterotomy + EPLBD	Failed extraction with balloon/basket catheters	33 33	31/33 (93.9%) 24/33 (72.7%)	0.021	3/33 (9.1%) 1/33 (3%)
